# Mechanistic Models of Protein Aggregation Across Length-Scales and Time-Scales: From the Test Tube to Neurodegenerative Disease

**DOI:** 10.3389/fnins.2022.909861

**Published:** 2022-06-30

**Authors:** Georg Meisl, Tuomas P. J. Knowles, David Klenerman

**Affiliations:** ^1^Yusuf Hamied Department of Chemistry, University of Cambridge, Cambridge, United Kingdom; ^2^Cavendish Laboratory, Department of Physics, University of Cambridge, Cambridge, United Kingdom; ^3^UK Dementia Research Institute, University of Cambridge, Cambridge, United Kingdom

**Keywords:** neurodegenerative disease, chemical kinetics, mechanistic models, protein aggregation, *in vivo* models, amyloid

## Abstract

Through advances in the past decades, the central role of aberrant protein aggregation has been established in many neurodegenerative diseases. Crucially, however, the molecular mechanisms that underlie aggregate proliferation in the brains of affected individuals are still only poorly understood. Under controlled *in vitro* conditions, significant progress has been made in elucidating the molecular mechanisms that take place during the assembly of purified protein molecules, through advances in both experimental methods and the theories used to analyse the resulting data. The determination of the aggregation mechanism for a variety of proteins revealed the importance of intermediate oligomeric species and of the interactions with promotors and inhibitors. Such mechanistic insights, if they can be achieved in a disease-relevant system, provide invaluable information to guide the design of potential cures to these devastating disorders. However, as experimental systems approach the situation present in real disease, their complexity increases substantially. Timescales increase from hours an aggregation reaction takes *in vitro*, to decades over which the process takes place in disease, and length-scales increase to the dimension of a human brain. Thus, molecular level mechanistic studies, like those that successfully determined mechanisms *in vitro*, have only been applied in a handful of living systems to date. If their application can be extended to further systems, including patient data, they promise powerful new insights. Here we present a review of the existing strategies to gain mechanistic insights into the molecular steps driving protein aggregation and discuss the obstacles and potential paths to achieving their application in disease. First, we review the experimental approaches and analysis techniques that are used to establish the aggregation mechanisms *in vitro* and the insights that have been gained from them. We then discuss how these approaches must be modified and adapted to be applicable *in vivo* and review the existing works that have successfully applied mechanistic analysis of protein aggregation in living systems. Finally, we present a broad mechanistic classification of *in vivo* systems and discuss what will be required to further our understanding of aggregate formation in living systems.

## 1. Introduction

Aggregated forms of normally soluble proteins have emerged as key structures in the pathology of a large number of neurodegenerative diseases, most notably Parkinson's disease (PD) and Alzheimer's disease (AD) (Chiti and Dobson, 2006; Eisenberg and Jucker, 2012; Knowles et al., 2014; Meisl et al., 2020b), but also in infectious prion diseases (Prusiner, 1982; Eigen, 1996). The appearance of aggregates is a common feature although the identity of the aggregating protein differs. For instance, in PD aggregates consist mainly of α-synuclein (Spillantini et al., 1997), while aggregates of the tau protein and of the Aβ peptide (or amyloid beta in its aggregated form) are both hallmarks of AD (Fitzpatrick et al., 2017; Yang et al., 2022). By contrast, prions are aggregates composed mostly of the PrP protein (Prusiner, 1991). Although the presence of protein plaques and tangles in the brains of affected individuals has long been identified as a hallmark of disease, work in recent decades has strengthened the evidence for a causative link between the presence of aggregates and pathology in several of these neurodegenerative diseases. In particular low molecular weight oligomeric intermediates have been recognized as particularly damaging to cellular function and many such soluble aggregate species are toxic in cell culture and cause inflammation. Furthermore, aggregate amounts in the brain correlate with the extent of atrophy, and mutations that increase the aggregation propensity or increase the concentration of the aggregation-prone protein often lead to earlier onset (Akiyama et al., 2000; Haass and Selkoe, 2007; Selkoe and Hardy, 2016; Chiti and Dobson, 2017). In the context of prion diseases, the role of protein aggregates, the prions, as causative agents of disease has been even more clearly established, with infection by prions being a sufficient condition for emergence of disease (Aguzzi, 2006).

To what extent the prion-like ability to effectively self-replicate and spread the disease-associated conformation is also present in other aggregation-related diseases has important implications for their management. Thus, the rate at which an aggregate is able to self-replicate is a fundamental characteristic of aggregating systems and can be a powerful way to quantitatively compare the aggregation in different systems. While in the context of self-replication, prions, and prion-like aggregates, behave similar to typical infectious agents, their underlying chemistry is remarkably simple: despite being normally soluble, aggregation-prone proteins can also exist in an alternate stable conformation, when associated with other proteins in a large, ordered aggregate. Formation of these aggregates in the test tube is often kinetically inhibited due to the energetic cost of nucleation of a new aggregate (Knowles et al., 2014). However, once a nucleus has been formed, further addition of proteins into the aggregate from solution is fast. In many systems existing aggregates can also trigger the formation of new aggregates by self-replication, altogether bypassing the need for further nucleation events once the first aggregated species are present (Knowles et al., 2011; Meisl et al., 2020b, 2022). This nucleated polymerization reaction can easily be reproduced in solutions of purified protein *in vitro* and has been shown to occur for many proteins (Meisl et al., 2016a), not only those linked to disease (Dobson et al., 2001; Fowler et al., 2007; Meisl et al., 2022).

The fact that the formation of these disease-associated aggregates is governed by this comparatively simple chemical reaction means the process lends itself to the application of powerful tools and techniques from physical and biophysical chemistry (Knowles et al., 2009). In particular, the framework of chemical kinetics offers a toolbox designed to answer key mechanistic questions. It allows quantification of the rates of different processes, to enable comparison between systems, as well as identification of the rate determining steps of a particular reaction. In this review, we will discuss the formation of prions and prion-like aggregates in neurodegenerative diseases from the perspective of molecular mechanisms. We initially outline the fundamental processes that make up different components of a mechanistic description of aggregate formation and then briefly discuss the strategies to establish mechanisms from *in vitro* data. We then discuss how these techniques can be applied in the analysis of data from living systems and conclude by providing our perspective on what will be needed to further our mechanistic understanding of neurodegenerative diseases.

## 2. Mechanisms and Rate-Limiting Steps in Complex Reaction Networks

A wide variety of different model systems are used to study different levels of complexity and aspects of aggregation related diseases, from mouse or cell models, down to studies of purified protein by methods of physical and biophysical chemistry. While these studies are usually able to answer well the very specific questions within their systems, progress is hampered by both, the difficulty of combining individual studies into a coherent picture and, more importantly, the challenge of translating the findings to human disease. This is partly due to the difficulty of extracting quantitative mechanistic parameters that can be compared across systems: the timescales involved differ by many orders of magnitude, from the hours needed to complete an aggregation reaction in the test tube to the decades it takes for the disease to progress through its stages in humans. As the techniques to monitor these experimental systems at a high temporal and spatial resolution and to modify them in a targeted manner are rapidly improving (Blennow et al., 2015; Ossenkoppele et al., 2016; Fitzpatrick and Saibil, 2019; Sang et al., 2021; Zimmermann et al., 2021), there is also a rapidly growing need for approaches that allow their analysis using biophysical models.

Both, a mechanistic interpretation and a quantification of the contribution of different processes to the overall behavior of the system are key for gaining a deeper understanding of aggregation phenomena across different systems and for driving rational drug development efforts. Chemical kinetics provides the toolset to achieve exactly this: a framework within which to formulate mechanistic hypotheses and then translate macroscopic observations into information about the microscopic mechanisms (Connors, 1990). A particularly powerful idea from chemical kinetics in the context of complex reaction networks, such as those encountered in aggregation in living systems, is that of a rate-determining, or rate-limiting, step. The idea is that, while a large variety of processes are occurring during the reaction, only one, or a handful of processes, actually control the overall behavior to a significant degree (Eigen, 1996; Meisl et al., 2017a). These processes are the control switches, the places at which interference significantly affects the system. Alterations to other processes have little to no effect on the overall behavior. Thus, identification of these processes can provide not only a more intuitive understanding of expected system behavior, but also point toward the most promising targets for pharmacological intervention (Bulawa et al., 2012; Arosio et al., 2014b). Much of the following will concern how the rate determining steps can be identified and show how different processes can be rate-determining in different systems.

## 3. The Fundamental Reaction Network of Aggregate Formation

**Figure 1:**
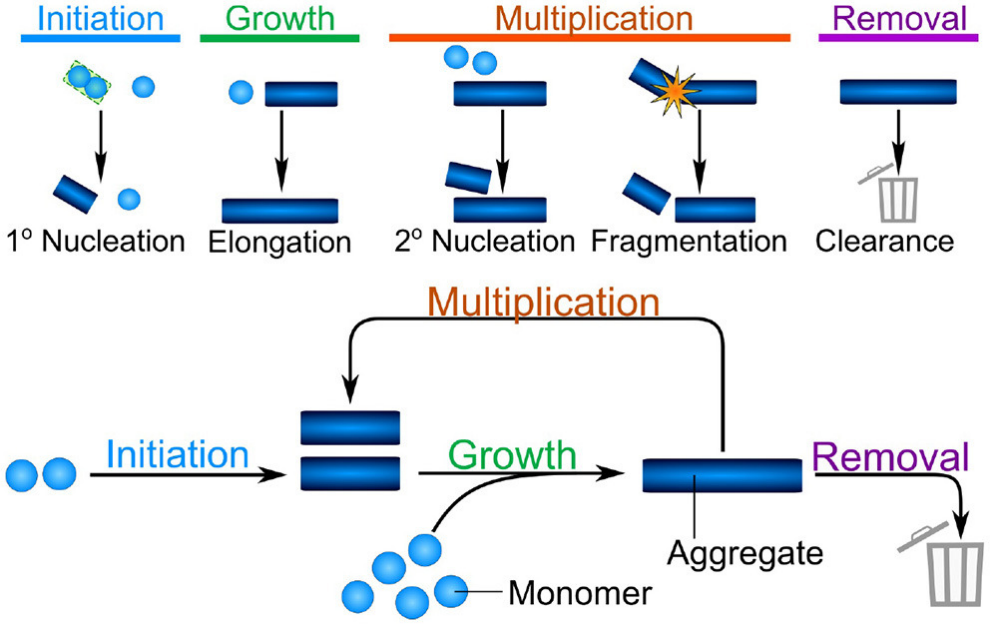
The different classes of processes combine into the fundamental reaction network of aggregation. Initiation is the formation of the first aggregates, without the involvement of existing aggregates. Growth is the increase in size of existing aggregates by addition of further protein subunits. Multiplication denotes any process by which existing aggregates trigger the formation of new aggregates, shown here schematically are fibril surface-catalyzed secondary nucleation and fragmentation. Finally, *in vivo*, aggregates can be removed by a number of clearance or degradation pathways. These fundamental classes of processes form the reaction network of aggregation, with growth and multiplication coupling together in a positive feedback loop, that gives rise to the characteristic exponential growth in aggregate mass that many systems display.

Despite the significant differences between the mechanisms of aggregate formation in different systems, such as the test tube and a living organism, a number of fundamental properties can be identified which will remain valid across systems. Indeed, four broad classes of processes can be defined: initiation, growth, multiplication, and removal (see Figure 1). *Initiation* refers to the formation of the first aggregates, without the involvement of any existing aggregates. This is a necessary first step in the aggregation of any purely monomeric system and can be the process that limits the rate of overall aggregate formation. In the context of some diseases, such as acquired prion diseases, this process is so slow that disease normally only arises when initiation is bypassed by the introduction of an initial aggregate, or seed, from an external source (e.g., another infected organism). *Growth* is the process by which existing aggregates increase in size. Once aggregates have formed, they can grow by addition of further proteins from solution, typically to sizes of several hundreds or thousands of proteins. *In vitro*, growth usually proceeds from the ends of linear aggregates, producing long fibrillar structures. *Multiplication* is the ability of existing aggregates to trigger the formation of new aggregates. Within this class we account for fundamental biophysical processes, which include the fragmentation of fibrils or the catalysis of nucleation on the surface of existing aggregates in the process of secondary nucleation. The latter in particular is a key mechanism of templated self-replication and interestingly represents a general process found in contexts beyond protein aggregation (Törnquist et al., 2018). In addition to these intrinsic means of multiplication, we also include indirect mechanisms which might be active in living systems, such as existing aggregates triggering a deleterious response in the organism that in turn triggers the formation of more aggregates. Multiplication is not necessary to completely aggregate a population of monomeric protein, however, it is a very common process. Finally, *removal* refers to all ways by which living systems can actively remove aggregates. This is the key fundamental difference in the reaction network of aggregate formation between the test tube and living systems and may be key in preventing run-away aggregation in organisms.

These broad classes of mechanisms can be found in relatively simple reactions in the aggregation of a purified protein *in vitro*, such as those shown schematically in Figure 1. As the complexity of the system increases, additional and more complex mechanisms will be coarse-grained into each of these classes. For example, initiation may involve attachment surfaces in a heterogeneous nucleation reaction, the formation of condensates, or interactions with lipid membranes. By coarse-graining these processes into the classes that make up the fundamental reaction network of aggregation, a key property is retained: the processes of growth and multiplication, when present, couple together in a positive feedback loop allowing aggregates to self-replicate. Thus, the presence of a multiplication step fundamentally changes the properties of the system: a few initiation events producing the initial fibrils can give rise to many child fibrils *via* self-replication, leading to exponential growth of the aggregate mass over time (Knowles et al., 2009; Cohen et al., 2011). Furthermore, one can bypass the slow initiation step by introducing a small number of preformed aggregates, significantly speeding up the reaction (Arosio et al., 2014a). By contrast, if aggregates are unable to multiply, each fibril has to be formed by a separate initiation event. This lack of positive feedback leads to a much more gradual increase of aggregate mass over time, and the introduction of pre-formed seed fibrils has little effect on the behavior of the system (Meisl et al., 2016a).

It is clear that the ability to self-replicate, both by providing a means to spread the aggregated conformation and by promoting rapid, exponential increase in aggregate amounts, might pre-dispose a protein toward involvement in disease (Törnquist et al., 2018). While little quantitative data exist on the self-replication of aggregates in living systems, the mechanism of aggregation of purified protein in the test tube has been determined for a large number of proteins. In Meisl et al. (2022) we analyzed a large number of published datasets within a chemical kinetics framework to quantify the contributions of the relative contributions of initiation and multiplication. These data show that the intrinsic ability to self-replicate, i.e., without the requirement for any cellular machinery, simply by means of fibril fragmentation or secondary nucleation, seems to be an almost ubiquitous property of protein aggregates. Remarkably, for those aggregates involved in disease, the intrinsic rate of self-replication is always fast enough to be relevant on a disease time-scale. Although these observations are based on test tube measurements, they nonetheless highlight that self-replication has to be considered as a mechanism of central importance across the range of aggregation-related disorders. To quantify this process, we define the doubling time, *t*_2_, which is the time taken for the number of aggregates to double. As self-replication leads to exponential growth in aggregate numbers, *t*_2_ is a constant and directly related to the rate of self-replication, κ, by the equation $t_{2}=\frac{\ln\left(2\right)}{\kappa }$. Typical doubling times range from under 1 hour (for μM test tube concentrations of Aβ42) (Cohen et al., 2013) to 5 years (for the accumulation of tau aggregates in the brains of Alzheimer's disease patients) (Meisl et al., 2021a). Doubling times provide a powerful and intuitive way to quantify the self-replication propensity in different systems.

## 4. Determining and Controlling Aggregation in the Test Tube

The aggregation of purified protein molecules under controlled *in vitro* conditions allows the intrinsic aggregation properties of proteins to be determined (Meisl et al., 2016a). The role of variations in protein sequence and solution conditions can be studied (Meisl et al., 2016b; Yang et al., 2018) and the effect of inhibitors and promotors of aggregation can be explored (Arosio et al., 2014b). Such experiments usually start with either a solution of purely monomeric protein, or monomeric protein with a well-defined amount of pre-formed aggregates added. Aggregation is usually induced by a temperature jump and then the accumulation of aggregates over time is monitored using a reporter of change in protein structure (dyes, intrinsic fluorescence), changes in turbidity, or imaging of aggregates by microscopy or other high-resolution techniques. Where time-resolved data is available, the variation of concentration of the different measured species over time can then be analyzed using chemical kinetics to determine mechanisms and extract rates. To do so, proposed mechanisms are turned into systems of differential equations describing the time evolution of the concentration of all reacting species using mass action. These equations are solved, in some cases approximately, to obtain integrated rate laws which can then be used to fit the data (Meisl et al., 2016a). The power of this approach stems from the fact that all the parameters determined in the fitting of these integrated rate laws have a physical meaning and link directly to the reaction mechanisms used to derive the rate law.

**Figure 2:**
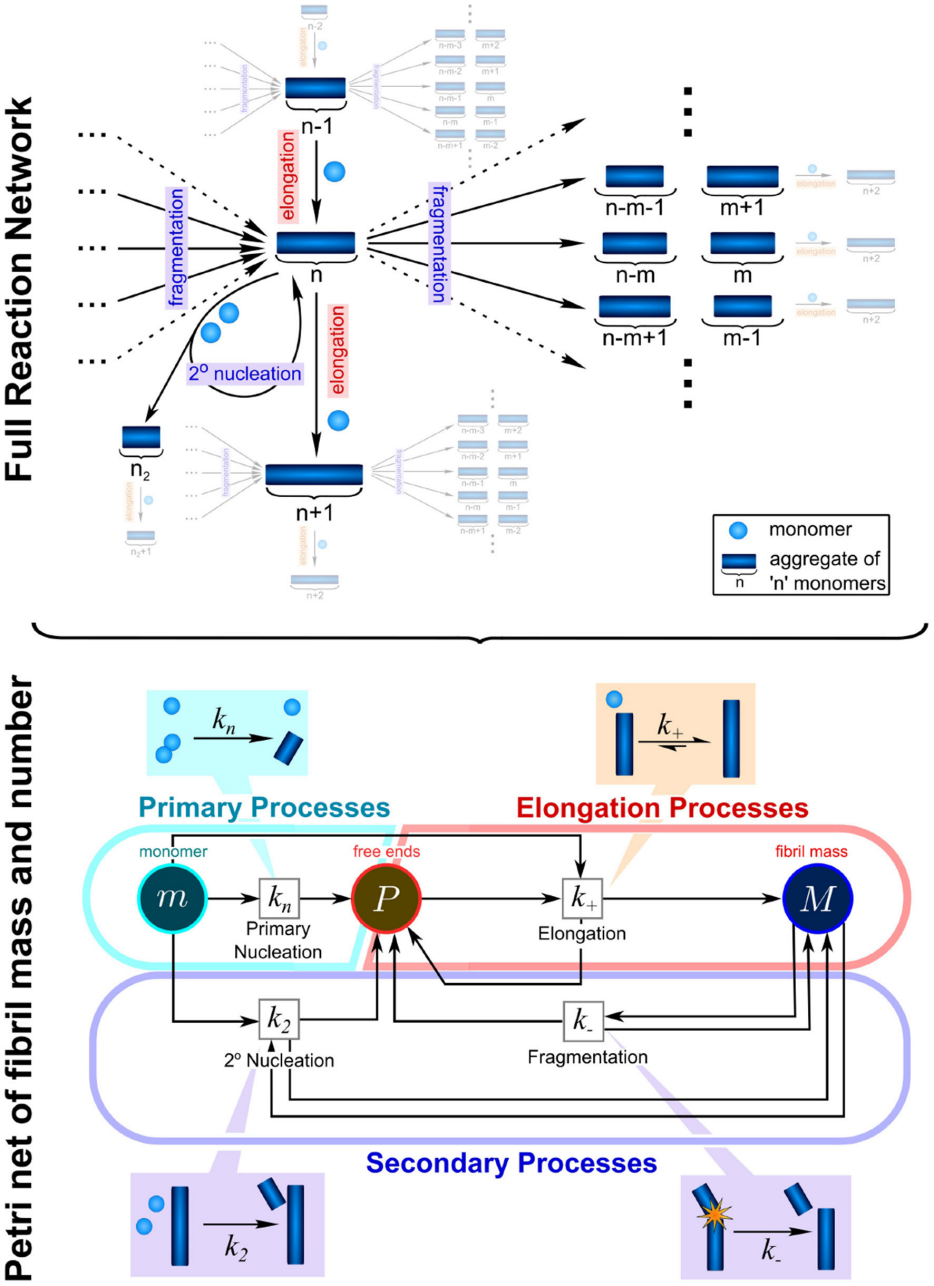
Complexity of reaction network can be significantly reduced by the introduction of moments. To fully describe the aggregation reaction one must account for how all the different sizes of aggregate can interact and inter-convert, leading to a highly complex reaction network with many thousands of possible species and reactions. This network can be enormously simplified by instead considering only the moments of the fibril length distribution, the aggregate number concentration, *P*, and the aggregate mass concentration, *M*. While some information about the detailed size distribution is lost in this step, the description of the system in terms of its moments is usually sufficient to fully describe most experimental data and to extract the rate constants of interest: *k*_*n*_, *k*_2_, *k*_−_, and *k*+. These are the rate constants of primary nucleation, secondary nucleation, fragmentation, and elongation, respectively. Adapted from Meisl et al. (2017a).

**Figure 3:**
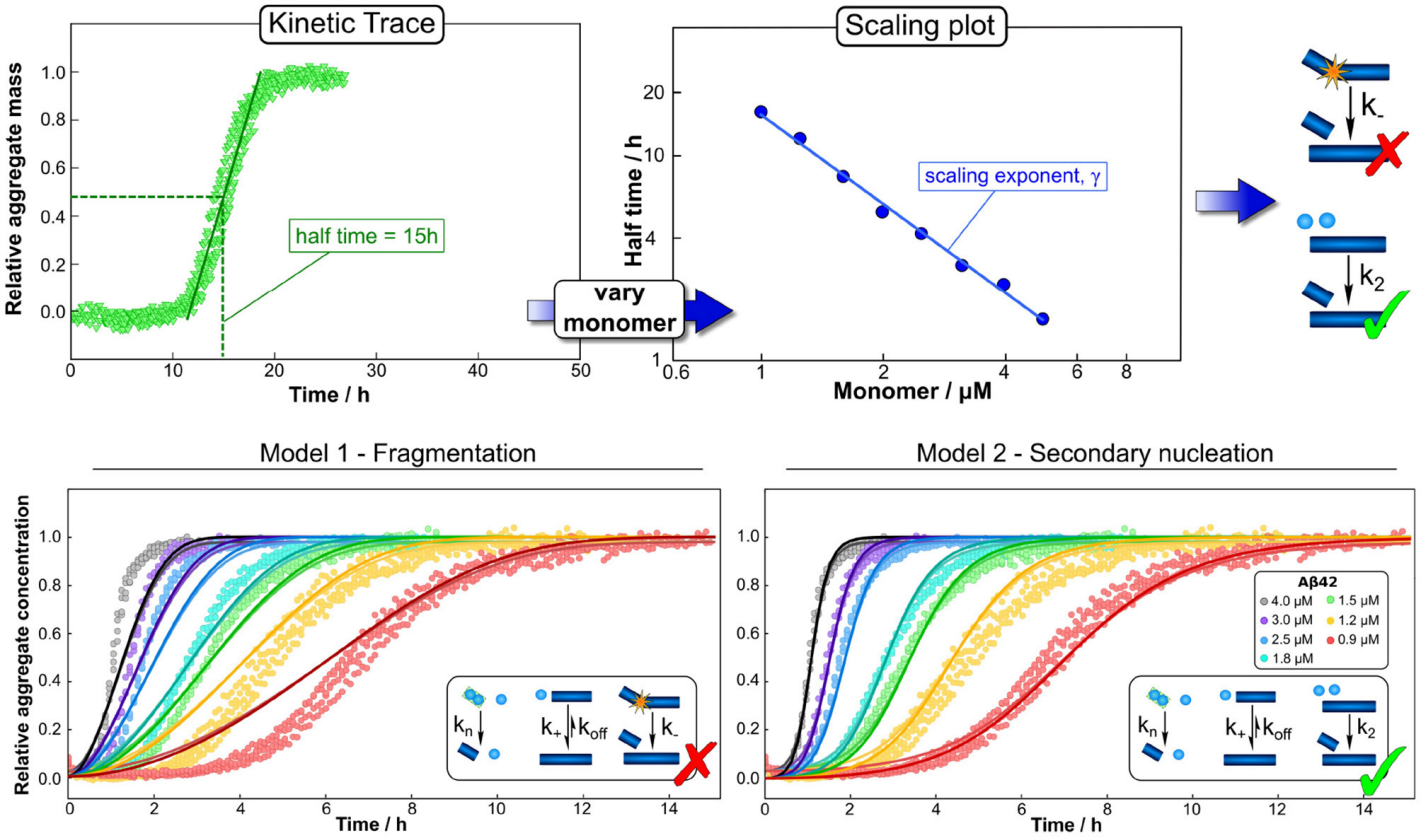
Global fitting and the use of scaling to determine mechanisms. Varying a number of different experimental parameters is crucial for determining possible mechanisms. In particular, variations of the monomeric protein concentration can provide important mechanistic insights due to the differing dependence of different mechanisms on monomer concentration. A powerful and easy way to apply this technique in practice is the use of scaling exponents, which describe how a representative quantity of the aggregation reaction, such as the half time, varies with the monomer concentration. **(Top)** The half time can easily be extracted from kinetic traces. Plotting the half time against monomer concentration then allows the determination of the scaling exponent and exclusion of mechanisms that are inconsistent with the observed scaling. Often scaling exponents alone already allow the qualitative determination of mechanisms (here an example in which fragmentation can be excluded simply based on the scaling exponent). **(Bottom)** To then quantify the rates and confirm mechanisms, one performs a global fit, i.e., using one set of parameters to describe the entire dataset at all monomer concentrations, of the integrated rate laws derived for different models (points are experimental measurements of aggregate amounts, solid lines the best global fit of the model shown in the schematic, different colors denote different protein concentrations). Here the fits confirm the conclusions from the scaling analysis, that a fragmentation dominated mechanism is inconsistent with the data while a secondary nucleation dominated mechanism describe it well. Adapted from Meisl et al. (2016a, 2017b).

In the context of protein aggregation, the rate law consists of a system of thousands of coupled differential equations, the master equation, which describe the population dynamics for each different aggregate size and type, from dimers to higher molecular weight species. Due to the non-linear nature of this equation integrated rate laws cannot easily be found (Figure 2). This difficulty can to some extent be overcome by coarse-graining the system to describe quantities that can be monitored experimentally: in this spirit, rather than focus on how all aggregate sizes evolve over time, one considers only the total number of aggregates and their average length, the first two moments of the aggregate length distribution. The resulting system of just two differential equations, the moment equations, is more tractable and fitting it to experimental data is in principle sufficient to determine the rates of initiation, growth, and multiplication (Knowles and Buehler, 2011). Beyond the derivation of mathematically accessible rate laws, the process of fitting itself also requires careful considerations of the data to be analyzed. The proposed mechanisms should be no more complex than required to fully describe the data and in turn the data should be acquired in such a way as to maximally constrain the possible mechanisms. Best results are thus achieved when experimental design and analysis go hand in hand, and one constantly informs the other. In practice this usually means that aggregation data should be acquired at different initial monomer concentrations and in the absence and presence of preformed seed fibrils. By analysing all those data together in a global fitting approach, one can obtain strong constraints on the possible models, despite the complexity of the underlying reaction network (Figure 3; Meisl et al., 2016a).

In addition to facilitating detailed global analyses, datasets that span a range of concentrations can give quick mechanistic insights through simple consideration of the concentration scaling of an easily accessible observable. In the aggregation of purified protein, such a quantity is the half time, the time at which half of the initially present monomeric protein has aggregated (Figure 3). How this half time scales with the initial monomer concentration is intricately linked to the underlying mechanism of multiplication. For example, a system in which aggregates multiply by fragmentation would show a different scaling from one in which they multiply by secondary nucleation on the fibril surface: the fragmentation process proceeds independently of any monomeric protein in solution, thus its rate does not depend on the monomer concentration. By contrast, secondary nucleation involves the coming together of several monomers on the fibril surface and therefore its rate will depend on the monomer concentration. Changes in rates result in changes in the half time and thus by determining how the half time changes with monomer concentration, one can determine whether aggregation proceeded *via* fragmentation or *via* secondary nucleation (Meisl et al., 2016a). A scaling analysis can even give detailed insights into more complex aspects of the reaction network, such as whether there are multiple processes competing in parallel or if a single process consists of several distinct sub-processes (Meisl et al., 2017a). Scaling laws are commonplace in many physical systems and in the context of protein aggregation they can be exploited for their robustness and easily accessible mechanistic information, both *in vitro* and in living systems, as we will demonstrate below. Finally, a crucial step in drawing mechanistic conclusions form experimental data is a careful interpretation of the obtained parameters in light of the coarse-graining of the model that will have necessarily taken place at the model selection stage. For example, the reaction orders of the nucleation reactions are, within the original derivation, interpreted as nucleus sizes and thus expected to take integer values. In practice, they can take non-integer values and are in fact unlikely to correspond directly to the number of proteins in the nucleus, instead describing the overall reaction order of a multi-step nucleation process (Meisl et al., 2014, 2016a). If part of the nucleation process takes place on interfaces, reaction orders can even approach 0 as interface saturation effects become important (Dear et al., 2020b). In many cases, the experimental data do not warrant explicit inclusion of individual steps and a coarse-grained single nucleation step is capable of fully describing the data (Dear et al., 2020a). If sufficiently detailed data are available, the system can be fine-grained to model more individual steps, but the level of coarse-graining always has to be adjusted to the system at hand and needs to be kept in mind during interpretation of the results.

Applying these techniques, the *in vitro* aggregation mechanism of a large variety of proteins has been determined in recent years: highlighting the correlation of aggregation rate and early onset in some disease-associated mutations (Flagmeier et al., 2016; Yang et al., 2018), describing the effect of solution conditions (Abelein et al., 2015; Meisl et al., 2016b), investigating the importance of surfaces in promoting aggregation (Galvagnion et al., 2015; Pham et al., 2016), and determining the roles of toxic oligomeric intermediates (Ludtmann et al., 2018; Dear et al., 2020c; Michaels et al., 2020). These analyses followed the strategy described above; first developing a mechanistic model from first principles, then deriving the integrated rate laws and finally global fitting to experimental data. However, the types of data analyzed and models used were diverse. Experimental data was obtained by different means, from single molecule measurements, over mass spectrometry, to the use of fluorescent dyes, such as thioflavin T, that report on total aggregate mass. Models ranged from the simplest ones only considering monomeric and fibrillar species, to those that explicitly include intermediate species and account for the presence of oligomers and their conversion between different states.

In the context of developing therapies for aggregation-related diseases, these techniques have also been extended to analyse the action of potential inhibitors of the aggregation process: depending on whether initiation, growth, or multiplication is affected, the addition of inhibitor has a characteristic effect on both the half time and the shape of the kinetic curves (Arosio et al., 2014b; Meisl et al., 2016a). This fact can be utilized to determine the mechanism of action of different compounds, which can then in turn be used to modify the aggregation reaction in specific ways. A wide range of compounds have been classified in this way, including Aducanumab, the first licensed disease-modifying drug against Alzheimer's disease, which was found to be an inhibitor of multiplication (Linse et al., 2020). Thus, *in vitro* mechanistic analysis provides a simple means for determining differences in intrinsic aggregation properties due to modifications of the protein and for screening for compounds that inhibit a specific step of aggregate formation.

## 5. Measuring Aggregation in Living Systems

Extending mechanistic analysis of aggregation phenomena to include *in vivo* measurements is crucial for progressing our understanding of the related disease. However, it is complicated by the difficulty of monitoring the concentrations of reacting species over time, as well as the increased complexity of the reaction itself, through interactions with other molecules present and also through the addition of a spatial component. The concentration of aggregates is generally low in living systems, thus necessitating highly sensitive methods. Furthermore, the aggregates can range in size and structure so ideally methods should also be able to address this heterogeneity. A comparison of a selection of methods is given in Table 1.

**Table 1:** Comparison of a selection of methods for *in vivo* measurements of aggregate concentrations.

**Method**	**Sensitivity**	**Type of aggregate**	**Spatial resolution**	**Longitudinal measurement**
ELISA	High	Most types	Medium (brain tissue)/	No (brain tissue)/
		(depending on antibody)	No (bodily fluids)	Yes (bodily fluids)
Seed amplification	Very high	Only replication-	Medium (brain tissue) /	No (brain tissue) /
(e.g., RT-QuIC)		competent	No (bodily fluids)	Yes (bodily fluids)
Super-resolution	High	Most types	Medium (brain tissue) /	No (brain tissue) /
Microscopy		(depending on reporter)	No (bodily fluids)	Yes (bodily fluids)
Structural	Low	Highly ordered structures	No	No
(e.g., cryo-EM)		(at atomic resolution)		
Histological stains	Medium	Large aggregates only	High	No
PET	Low	Most types	Medium	Yes

*PET stands out as the only method that is able to record truly longitudinal data of aggregate distributions in the brain; all other methods have to make the choice between spatial resolution or longitudinal measurements. Seed amplification techniques distinguish themselves by being highly sensitive (potentially down to single seed level), but are selective to only those aggregates that are replication-competent*.

ELISA-based methods with sufficient sensitivity to detect the overall aggregate concentration and follow disease progression have been developed, but provide little spatial and structural resolution (Mattsson et al., 2009; Savage et al., 2014; Yang et al., 2015). Other highly sensitive amplification-based methods have been developed to quantify the number of aggregates present using a test tube or cellular seeding assay (Klohn et al., 2003; Atarashi et al., 2007; Holmes et al., 2014; Groveman et al., 2018). Those methods utilize the propensity of the disease-related aggregates to self-replicate by designing a reporter system that amplifies even minuscule concentrations of aggregates in a sample to detectable levels. These ideas originally found application in prion research (and before that in virology; Reed and Muench, 1938) where the infectivity of a sample was determined by inoculation of test animals with serial dilutions of the sample until it no longer caused disease. These measurements are particularly relevant for the mechanistic analysis of systems where self-replication of aggregates plays a central role in the mechanism as they, by design, only detect those species that are able to self-replicate. The readout of these methods is not affected by the presence of any inert aggregates, which are not involved in the reaction and thus should not be considered as part of the self-replicating aggregate population in the modeling. While these techniques can be highly sensitive, they suffer from potential problems that the method used to process the brain tissue, often homogenization, may break up larger insoluble aggregates into smaller seeds and less perturbative methods might be better to extract the aggregates such as soaking (Hong et al., 2018). Furthermore, there are often no longitudinal data monitoring the same patient over time (in the case of post-mortem measurements), nor is there information on the spatial distribution of aggregates in the brain (when bodily fluids are used).

In order to obtain spatial information, a range of imaging methods exist. Histology and *in situ* methods, using dyes or antibodies to detect aggregates, can provide spatial resolution down to a cellular level, and are used as standard ways to classify the stage of disease progression (Braak and Braak, 1991). However, they are generally not as quantitative as the techniques described above, and may be biased toward large aggregate clusters in the form of plaques or tangles (DeVos et al., 2018). More recently, single molecule fluorescence has been shown to have sufficient sensitivity to detect single aggregates and when combined with super-resolution imaging can obtain information about aggregate size and structure (Whiten et al., 2018; De et al., 2019; Zimmermann et al., 2021). While these techniques can obtain better spatial resolution than ELISA- or amplification-based methods, they still generally suffer from the drawback that no longitudinal data are available.

To address this issue, in recent years it has become possible to quantify the amounts of aggregated tau and Aβ aggregates directly in living patients using PET imaging. Although these measurements are significantly less accurate than the post-mortem measurements discussed above, they can be used to obtain longitudinal datasets that quantify how the amounts of aggregated species evolve over the course of the disease in individuals (Sanchez et al., 2021). With both temporal and spatial resolution of how aggregates accumulate, the enormous potential of these datasets to give key insights highlights the need for frameworks to analyse them with mechanistic models to complement current analysis strategies.

## 6. Approaches to Modeling Aggregation in Living Systems

The major focus of mathematical modeling has been on the analysis of patient data in the context of determining correlations and connections between different measurements and different aspects of the disease. A hallmark of many neurodegenerative disorders is a characteristic spatial evolution of the disease, leading to the classification into successive disease stages, based on the areas of the brain that are affected by pathology (Braak and Braak, 1991). A key area of modeling is thus concerned with these spatial patterns, in particular how they distinguish different diseases and which parameters, such as neuronal connectivity or selective vulnerability, drive the appearance of these patterns (Cope et al., 2018). Many of these current strategies use a coarse-grained model of the interconnected system of neurons, based on detailed brain connectivity data. It should, however, be noted that there is still some debate on the mechanisms that allow aggregates to spread through the brain in different diseases, including *via* tunneling nanotubes (Tardivel et al., 2016) or involving microglia (Pascoal et al., 2021). Such connectivity-based modeling methods have for example discovered disease-typical spatial patterns (Weickenmeier et al., 2018) and found a clear link between the appearance of Aβ aggregates and pathological tau in Alzheimer's disease (Vogel et al., 2020). The core focus of these models is on finding macroscopic correlations and connections, but there are some recent works to link them to mechanistic models of aggregation at the molecular level (Fornari et al., 2019; Meisl et al., 2021a,b).

Generally, when it comes to designing mechanistic models of aggregation in living systems, a number of factors fundamentally alter the behavior from what one might expect *in vitro*: (1) Due to protein synthesis and degradation, total protein concentrations are no longer constant as they are *in vitro*. (2) Modifiers of aggregation, such as chaperones or the presence of lipid interfaces, alter the rates of the different classes of processes; initiation, growth, and multiplication. (3) While *in vitro* systems are generally well-mixed and homogeneous, confinement and the differences in environment between individual cells and different brain regions introduce a spatial component. (4) There are process which actively remove aggregates from the reaction, such as clearance through autophagy and export from cells.

The first two factors can be addressed by minor alterations to the reaction networks used *in vitro*. Inhibitors or promotors of aggregation can be well-modeled as perturbative effects (Arosio et al., 2014b; Galvagnion et al., 2015) and different conservation laws can be enforced to account for monomer production and removal processes (Nowak et al., 1998; Poeschel et al., 2003; Meisl et al., 2020a). Post-translational modifications, such as phosphorylation, nitration, and truncation, could also be included in this group. However, unlike the other effects discussed here, the discrepancy between *in vivo* and *in vitro* behavior in that case does not stem from any increased complexity of the *in vivo* reaction network, but simply from the fact that the properties of the aggregating proteins differ. If the difficulty of obtaining the correctly modified protein in sufficient quantities for *in vitro* experiments can be overcome, this problem can be avoided.

By contrast, spatial inhomogeneities can have a more significant effect as transport rates can play a significant role in determining the overall behavior (Fornari et al., 2019; Meisl et al., 2021a). During modeling, the ordinary differential equations that are used to describe the reaction in well-mixed systems may have to be replaced by partial differential reaction diffusion equations to account for these spatial inhomogeneities. The introduction of a spatial dimension also poses a key question in the context of disease progression: how important is the spreading of aggregates throughout the organism, compared to their *de novo* formation and self-replication? Establishing which of these processes dominates disease progression is thus essential for targeting the correct steps in the rational search for therapies. An example of how this question can be addressed is given below in the Section 7.4.

Finally, the presence of aggregate removal or clearance processes has the potential to cause the most drastic difference of the aggregation behavior compared to the situation in the test tube; it allows systems to be in a stable state, constantly producing aggregates without ever entering a runaway aggregation regime (Thompson et al., 2021). One can determine a critical clearance rate: if aggregates are cleared at a rate faster than this, the system is stable, and there is no runaway aggregation. If, for some reason, the clearance rate is decreased below this critical value however, the system switches to runaway aggregation. This finding highlights the potentially central role of the clearance process in the emergence of disease, in particular in light of the finding that protein quality control mechanisms decline with age (Labbadia and Morimoto, 2015).

## 7. Mechanistic Analysis in Specific Systems from Cell Culture to Patient Data

Initial progress made in the mechanistic analysis of data from living systems shows promising insights, which we briefly outline here in systems of increasing complexity. While the specifics will differ from system to system, the crucial questions generally revolve around which process or processes are rate-limiting for the overall process and how their rates compare to that of the same protein aggregating under different conditions, such as in its purified form *in vitro*.

### 7.1. Cellular Removal Processes and Seeding

In the context of studying aggregation mechanisms, experiments in cell culture have been key for investigating the factors that might influence propagation, such as exploring how seeds enter into the cells. For example, in the context of tau aggregation, it was found that aggregates themselves are taken up by cells and are then able to induce the conversion of more monomeric tau inside the cell into its aggregated form, in analogy to how seeding proceeds *in vitro* (Frost et al., 2009). Far fewer studies have focused on the mechanisms by which seeds then trigger aggregation and by which the cell can combat aggregate accumulation, since answering these questions requires still more sensitive and quantitative measurements. Super-resolution imaging offers a way to follow the accumulation of different types of aggregates, both within the cell and in the cell medium. Employing this technique to monitor α-synuclein aggregation in SH-SY5Y cells, seeding was found to be a very inefficient process, requiring high levels of seeds to overcome protective cellular mechanisms (Sang et al., 2021). Similarly, in an organotypic slice model of tau seeding, it was found that no significant seeding was observed even at concentrations of tau higher than physiological levels (Miller et al., 2021). Remarkably, in this organotypic slice culture, tau seeding efficiency scaled more strongly than linear with tau seed concentration, implying that multiple tau seed aggregates were needed for seeding. It is, however, difficult to directly translate these findings into human disease, given the use of recombinant seeds, tau over-expression, and the uncertainty about what other factors may alter the efficiency of the seeding process in the diseased brain. Nonetheless, these studies show that seeding is an inefficient process in slice and cell culture and that multiple aggregates may need to stress a single cell in order to overcome its protective mechanisms.

Once seeding occurs, aggregate formation inside the cell may be a comparably fast process. In SH-SY5Y cells aggregate formation upon successful seeding was found to be a rapid process with a doubling time of 5 h, orders of magnitude faster than in the test-tube (Sang et al., 2021). Remarkably, in this system, blocking proteasome activity revealed that the proteasome was responsible for increasing the rate of aggregation. This is likely to occur *via* an increase in the rate of fragmentation by at least 10-fold, as has been observed previously (Cliffe et al., 2019). By contrast, small aggregates (~35 nm long) of both Aβ and α-synuclein were found to be secreted continuously into the media and this export increased upon seeding. Unlike the proteasome, secretion, therefore, appears to be an important protective process for aggregate removal. These observations highlight that aggregate degradation and removal is likely to be a complex process involving several distinct mechanisms. In addition to secretion and degradation, autophagy is likely to be a key process employed by organisms to combat protein aggregation (Nixon, 2007; Rubinsztein et al., 2015) and as such may find application in therapy (Menzies et al., 2017; Uddin et al., 2018).

### 7.2. Initiation Events and Stochasticity

**Figure 4:**
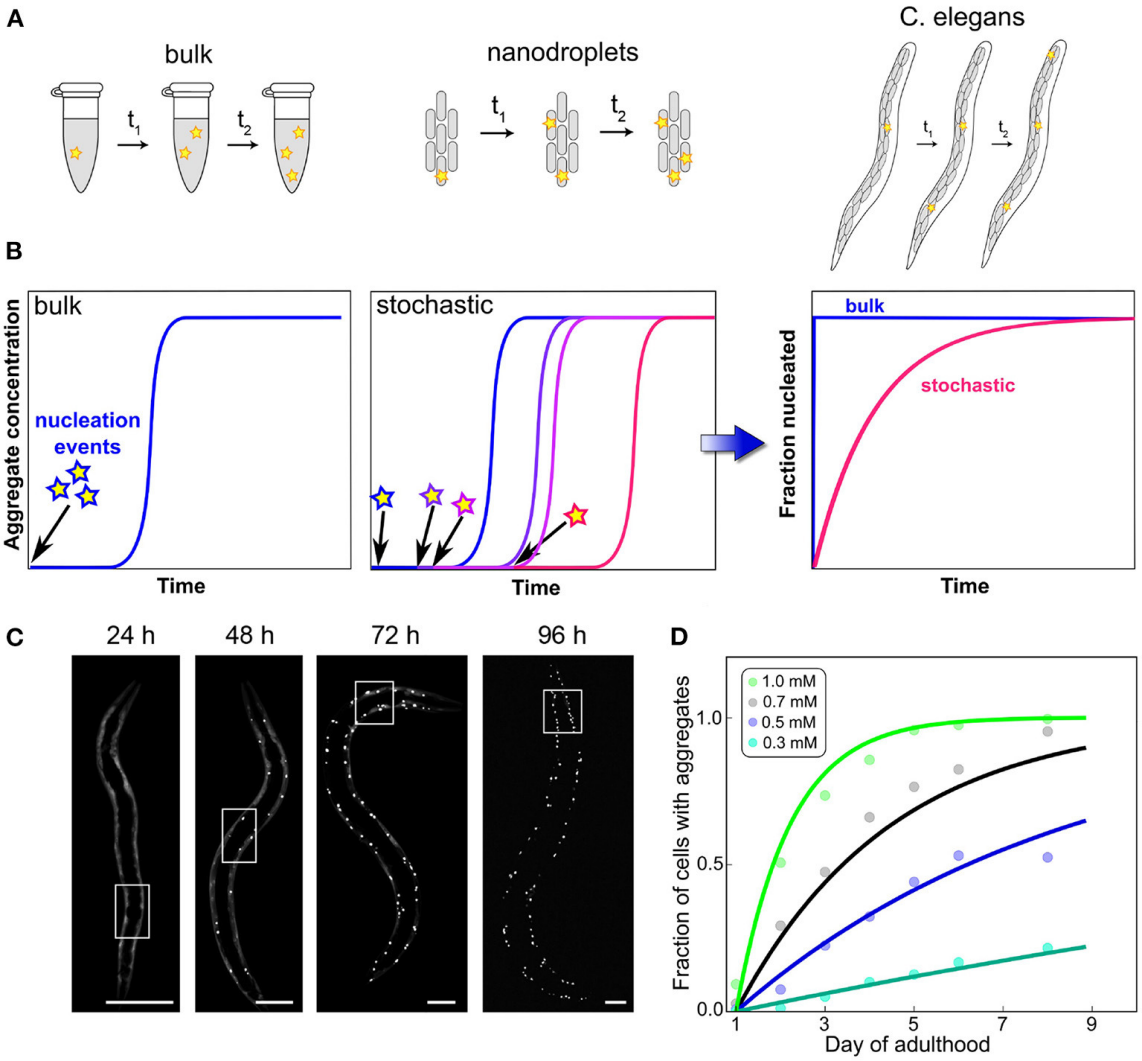
Confinement and determination of the kinetics by a stochastic nucleation event. In bulk, many initiation events take place in a short amount of time, compared to the overall timescale of the reaction **(A)**. This leads to reproducible curves **(B)** and can often mean that little information about the initiation process can be obtained from an analysis of the data because they are dominated by other processes. By contrast, when the same reaction is carried out in volumes so small that initiation is rare on the timescale of the aggregation reaction, stochastic behavior emerges. Each experimental curve is governed not only by the rate constants of aggregation but also by when the random event of nucleus formation occurred. This effect can also be observed in the aggregation of polyQ in *C. elegans* worms (scale bars 50 μm), where each cell behaves like an independent reaction vessel **(C)**. By globally fitting how the fraction of aggregated cells varies over time for different polyQ concentrations, the reaction order of the initiation process can be determined **(D)**. Adapted from Sinnige et al. (2021).

While cell culture can thus answer questions surrounding clearance by export and the susceptibility to aggregation induced by seeds introduced to the cellular medium, multicellular model systems can also investigate intercellular interactions. In particular, the question whether initiation happens in a cell-autonomous manner or whether aggregation in one cell can induce aggregation in neighboring cells is important for understanding the emergence of disease in multicellular organisms. Sinnige et al. (2021) studied this question in the context of polyQ aggregate formation in *C. elegans*, as a model system for Huntington's disease. Monitoring the aggregation by imaging methods, they found that initiation proceeds in a cell-independent manner and that the rate-limiting step is the initiation of aggregation in a given cell. In other words, in this system cells behave as independent reaction vesicles, with only the nucleation step controlling the appearance of aggregates. Once nucleation has occurred, the cell rapidly reaches its fully aggregated state, but nucleation events are rare and therefore the system contains mostly non-aggregated or fully aggregated cells (Figure 4). This simple behavior resembles that of a set of confined *in vitro* volumes, as for example in aggregation reactions carried out in microdroplets (Knowles et al., 2011). Remarkably, the reaction orders determined in *C. elegans* for polyQ parallel those determined for the purified protein *in vitro*. These systems highlight the special role that the initiation event can play: when growth and multiplication are fast enough, the behavior of the entire cell can be governed by a single, stochastic molecular event, the formation of the first aggregate. Such a behavior is only possible when initiation is so rare that on average only a single event occurs over a typical aggregation timescale. Its hallmark is a significant variation in the kinetics of equivalent repeats of the experiment, due to the inherently random nature of the process that dominates the kinetics (Michaels et al., 2018). Thus, stochastic nucleation cannot generally be observed in bulk *in vitro* reactions where many nucleation events take place. The “lag time” in those typical bulk aggregation experiments should therefore not be misinterpreted as a waiting time for nucleation to occur. As has been exhaustively demonstrated, the lag time in bulk aggregation reactions is simply the time until a sufficient quantity of aggregated material has accumulated to be detected and the sudden increase of the curves is merely a property of the expected exponential increase in aggregate amounts (Arosio et al., 2015).

The degree to which confinement into individual cells dominates the overall behavior will depend on the specifics of the system, such as how the rate of initiation compares to the rate at which aggregates formed in one cell induce the formation of aggregates in another cell. The above is an example of an extreme situation where the initiation dominates. By contrast, acquired prion disease in which all aggregates stem from those introduced upon infection, is an example of the opposite extreme. As with the other general classes of processes we have introduced here, it is important to remember that initiation itself is the coarse-graining of potentially several molecular steps which together result in the formation of the first self-replication-competent aggregate (Dear et al., 2020a). Initiation in particular may involve interactions with surfaces, such as lipid membranes which are believed to play an important role in the initiation of α-synuclein aggregation (Galvagnion et al., 2015), or the formation of condensates by liquid-liquid phase separation (Babinchak and Surewicz, 2020).

### 7.3. Scaling and Detailed Mechanisms

**Figure 5:**
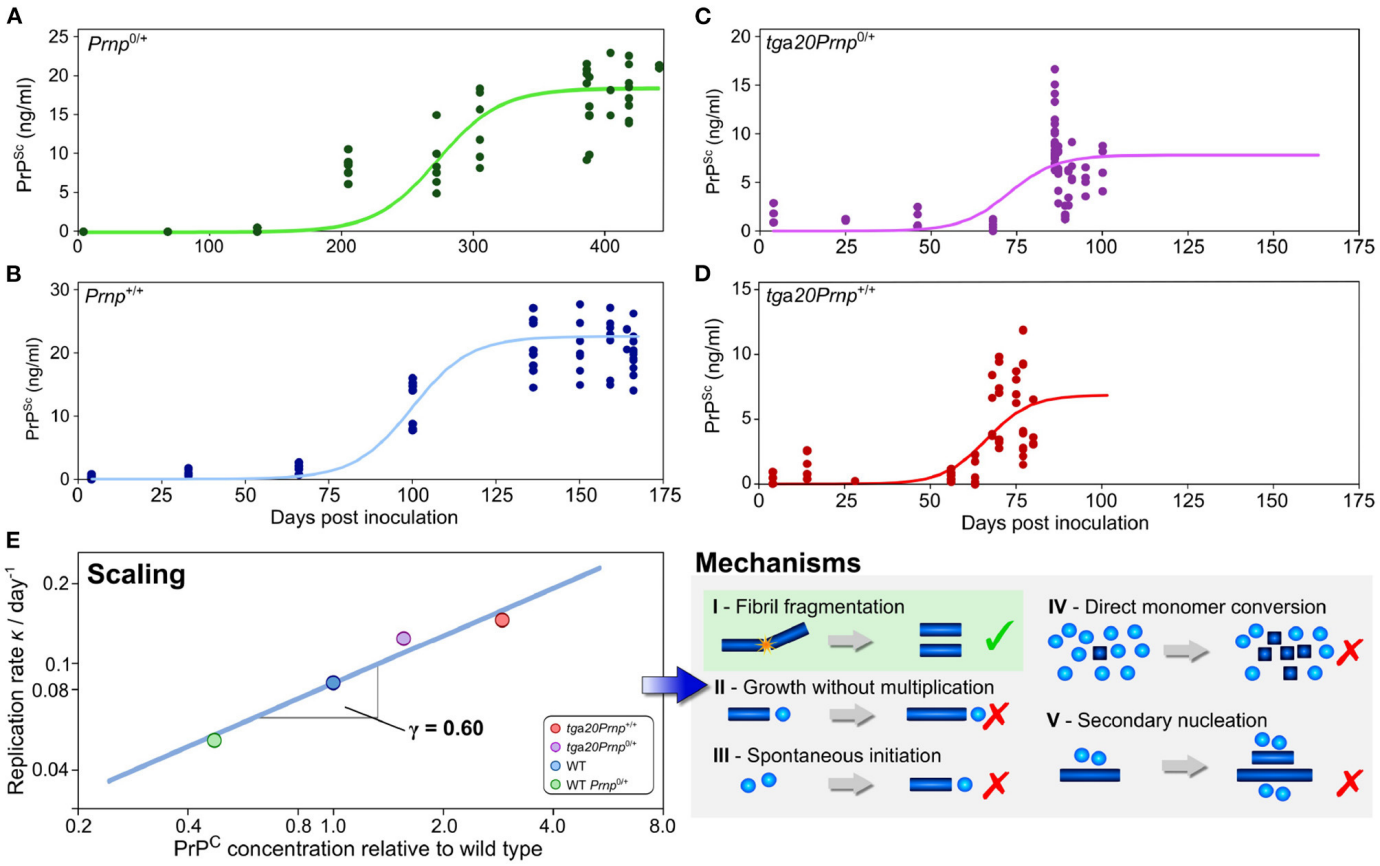
Use of scaling analysis to determine the mechanism of replication in a living system. The concentration of prions was determined as a function of time after inoculation by prions in a number of different mouse lines, with technical and biological replicates shown as solid points **(A–D)**. The mouse lines express the different amounts of the monomeric precursor, PrP, as shown in **(E)** relative to the wild type line *Prnp*^+/+^. The rate of replication is extracted from those data by fitting of a minimal model (solid lines). In turn, the variation of the replication rate with the concentration of the precursor protein PrP allows one to determine the scaling exponent, γ = 0.6 **(E)**. This low scaling, with the rate increasing approximately with the square root of the protein concentration, allows a range of mechanisms to be excluded and is consistent with the mechanism of prion multiplication determined for the purified protein *in vitro*. Adapted from Meisl et al. (2021b).

As has been outlined above, the scaling of the half time, or equivalently of the reaction rate, with protein concentration can help narrow down possible mechanisms of aggregation *in vitro* (Meisl et al., 2017a). When sufficiently detailed data are available, such an analysis can also be carried out *in vivo*, although the situation is complicated by the fact that there are now a much larger number of reasonable reaction mechanisms. This can be addressed by a careful consideration of the fundamental requirements on aggregation reactions *in vivo* in order to obtain a set of classes of minimal models (Meisl et al., 2020a). While a scaling analysis will then not produce a single reaction mechanism at molecular resolution, it will be able to exclude entire classes of mechanisms that are inconsistent with the data. The application of this technique is demonstrated in Meisl et al. (2021b), where the mechanism of prion multiplication was determined in mice. Four strains of mice that express the monomeric prion precursor protein (PrP) at different levels were inoculated with prions. The concentration of prions was then determined at different timepoints throughout the disease. As the assay requires the post-mortem determination of prions in brain homogenate, a pseudo-timecourse has to be pieced together by combining measurements from different mice at a number of timepoints after inoculation. Obtaining a time-evolution of the concentration of aggregated species is crucial for mechanistic analysis, thus, when only post-mortem measurements are available, they need to be combined into a representative time-trace. From the reconstructed time-traces, the rate of self-replication can be determined through fitting and the scaling of this rate with monomer concentration can be calculated (Figure 5). In the case of prions in mice, it was found that the rate of self-replication scales approximately with the square root of the protein concentration. This scaling is consistent with the class of mechanisms that include formation of linear aggregates that multiply by fragmentation and allows exclusion of the originally proposed hetero-dimer mechanism as the mechanism of multiplication. Fragmentation of linear fibrils has also been established as the mechanism of aggregation of purified PrP *in vitro*, indicating that the *in vitro* and *in vivo* mechanisms may be surprisingly similar. Despite this potential similarity in mechanism, a determination of the rate of self-replication shows that it is slowed down significantly in the mouse compared to the purified protein in the test tube. These findings demonstrate the power of scaling analyses in the determination of aggregation mechanisms and the importance of obtaining data under a range of conditions, for example at different concentrations of the monomeric precursor.

### 7.4. Spatial Factors and Human Data

**Figure 6:**
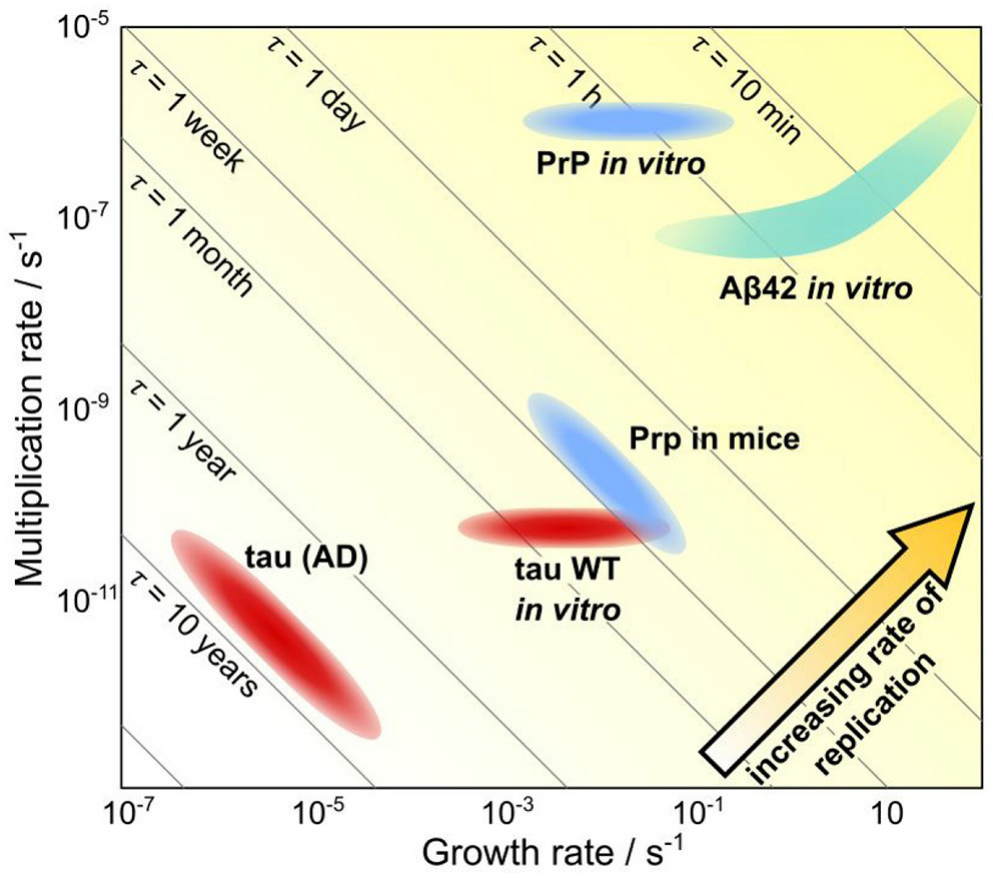
Quantification of timescales of aggregation across systems. A plot showing the growth rate versus the multiplication rate, for a number of proteins, using either rates determined *in vivo* or rates determined *in vitro* and extrapolated to *in vivo* concentrations. Along the diagonal lines, the doubling time is unchanged, thus going from the bottom left hand to the top right hand corner the rate of replication increases. Notably, the same proteins tend to aggregate orders of magnitude slower *in vivo* than they do *in vitro*, highlighting the ability of living systems to prevent aggregation. Adapted from Meisl et al. (2021a).

Mechanistic analysis of human data is even further complicated beyond that of animal model systems since no control can be exerted over factors such as the monomer concentration and that longitudinal data of neurodegenerative diseases is challenging to obtain. Thus, the questions being asked and models being used in analysis must be adjusted accordingly. At the top level lies the determination of the relative importance of the different fundamental classes of processes; initiation, self-replication and spreading through space. We were able to demonstrate this approach in a recent work by using data from patients to determine the rate-limiting step for the formation of tau aggregates in the later stages of Alzheimer's disease (Meisl et al., 2021a). The spatial evolution of aggregate concentration forms the basis for staging AD into Braak stages and thus the process of spreading has received a large amount of attention. However, we found that in the mid to late disease stages (Braak stage III onwards) the rate-determining step was the local self-replication of aggregates, rather than spreading between brain regions. Through modeling we were able to show that changes in the rate of spreading between brain regions are expected to have very little effect on overall progression. As in other systems where the rate of self-replication has been determined, the doubling time observed *in vivo* is orders of magnitude longer than that observed for the purified protein *in vitro*, hinting that there are powerful mechanisms to prevent aggregate accumulation at play *in vivo* (Figure 6). This finding is also supported by the effect of bodily fluids on slowing the aggregation of purified protein in the test tube (Frankel et al., 2019). The analysis of AD data demonstrates that even a top level analysis with models coarse-grained into only the very fundamental classes of processes can provide new mechanistic insights and a means to quantitatively compare different systems.

### 7.5. Strains and Sporadic vs. Acquired Diseases

A question of major importance is how aggregates appear throughout the brain in early disease. One can imagine two limiting behaviors: a prion-like spreading mechanism where one or a small number of initial aggregates replicate exponentially and spread through the brain, and a direct initiation mechanism where aggregates form spontaneously in individual cells. Most real systems may fall somewhere between these two limits, and the behavior of different aggregate strains may provide information on the dominant process. Elegant cryo-electron microscopy experiments have shown that the aggregates formed in different diseases have distinct structures while people with the same disease have aggregates with the same structure (Fitzpatrick et al., 2017; Falcon et al., 2018; Zhang et al., 2019). If the type of strain determines the type of disease, this observation of distinct strains in distinct diseases would imply that single initiation or infection events, followed by prion-like spreading are dominant. However, no such conclusion can be drawn if instead there are other factors (genetic or environmental) that determine the disease, which in turn determines the strain. If the type of disease determines the propensity of different strains to form, the formation of many new aggregates directly, throughout susceptible areas of the brain, is equally consistent with the observation of strains as a single initiation event from which prion-like spreading occurs. While for acquired diseases, there is little doubt about the dominance of prion-like mechanisms, in spontaneous disease, the observation of distinct fibril structures in different diseases does not allow one to distinguish between the two limiting mechanisms until the causal relation between fibril strain and disease type can be established.

## 8. Route Forward, Open Questions, and Key Unknowns

While we have demonstrated a range of techniques and systems in which it has been possible to gain new mechanistic insights, many big questions surrounding the aggregation-related neurodegenerative diseases remain, particularly in humans. To help structure the discussion, we provide here a way to classify different questions and hypotheses.

The key questions about disease mechanisms can be separated into two main categories of “How does aggregate formation begin?” and “What controls its proliferation?.” In most cases very little is known about the crucial initiation events, the triggers that start aggregation. To classify the different hypothesis we again propose the consideration of two extremes: initiation is independent of changes in external factors or initiation is triggered by changes in external factors. We use the term *external factors* here to signify any factor that changes over time other than the formation of aggregates, for example decline in protein quality control, infection etc. We choose this classification for its utility in disease prevention as such external triggers can serve as potential targets if they are important in initiation. Systems in which initiation is independent of external factors, as we define them here, would be the aggregation of purified protein in a test tube (initiation happens at the beginning of the reaction, aggregates build up over time, and the only relevant timescale is that of aggregate formation) or the appearance of polyQ aggregates in the cells of *C. elegans* worms as discussed above (initiation is a random event, whose probability is constant over time). In other words, models in which aggregation proceeds gradually, with unchanged rate constants, over the entire life of the organism, or in which it is triggered by a random nucleation event, fall into this category. Systems which we would classify as triggered by external factors are any that involve infection, such as acquired prion disease (aggregation is initiated by the introduction of an infectious prion), but also those proposed models in which bacterial infections trigger neurodegenerative disease, or in which inflammation plays a causative role (Lue et al., 1996; Akiyama et al., 2000; Rietdijk et al., 2017; Kinney et al., 2018; Dominy et al., 2019). In the same category, but somewhat distinct, are those external factors linked to ageing, such as the decline in protein quality control mechanisms (Labbadia and Morimoto, 2015). If for a disease the age of onset is relatively conserved and potentially correlated with external factors, initiation by a single stochastic event is unlikely. If a purely random event is the cause of initiation, the variance of the time of initiation should be comparable to the mean time of initiation, which is inconsistent with a conserved age of onset (we expect an approximately exponential distribution of initiation events over time, as in the case of *C. elegans* discussed above; Sinnige et al., 2021). The fact that most neurodegenerative diseases are diseases of old age suggests that ageing-related factors play a significant role in determining age of onset, suggesting that external factors, as we define them here, play at least some part in determining initiation.

The change in external factors also allows for a new type of initiation that cannot be produced in this manner *in vitro*. In this scenario, the initiation event is not the appearance of the first aggregates, but rather a switch from a stable state, in which aggregates are being removed as quickly as they are formed, to one in which clearance can no longer keep up with aggregate formation and thus runaway aggregation occurs. Since the system is initially in a stable steady state, an external change in parameters, such as a decrease in the clearance rate, is necessary to trigger the switch (Thompson et al., 2021). When it comes to controlling such systems, one can either attempt to inhibit the aggregation process or accelerate the removal process. Quantification of the rates of both processes would be key in determining the critical rate at which the switch from run-away aggregation to a stable state can be achieved.

Somewhat separate from the question of initiation is the question of progression. As previously demonstrated (Meisl et al., 2021a), to understand the overall effect on progression, it will be crucial to quantify the rates of both self-replication and spreading in order to determine which contribution dominates the behavior. In order to judge the importance of spreading compared to self-replication, spatial inhomogeneities have to be taken into account. Two questions are important in the consideration of spatial inhomogeneities: (1) how existing aggregates in one place trigger the formation of aggregates in a different location, for example by transport along neuronal connections or involvement of microglia. (2) how reaction rates, such as the propensity for aggregate formation, vary across brain regions. Dissecting which process plays a dominant role, can, for example, help determine if particular regions are intrinsically protected or vulnerable, or simply less or more affected by transport from neighboring brain regions.

In principle aggregate formation can proceed without self-replication, but the overwhelming evidence is that self-replication, in one form or another, occurs in the majority of aggregation-related diseases, in particular AD (Clavaguera et al., 2013; Meisl et al., 2021a). While spatial inhomogeneities and spreading can affect the overall rate of progression in some situations, self-replication always does (Meisl et al., 2021a). The question of progression is then how existing aggregates trigger the formation of additional aggregates. The mechanisms of self-replication can again be separated into those that are direct, likely paralleling those that are present *in vitro*, and those that are indirect, such as stresses exerted by the presence of aggregates that trigger aggregation within the stressed cells. Identifying the type of self-replication mechanism that dominates the overall rate can thus inform on potential targets for therapeutic intervention.

The application of these ideas to answer key questions in neurodegenerative disease from patient data will require the combination of sophisticated mechanistic models with cutting-edge analysis strategies. In all cases, a crucial component to successfully develop our mechanistic understanding will be the accurate measurements of aggregate concentrations and compositions, resolved both in space and most crucially over the course of the disease. A deeper quantitative and mechanistic understanding will enable the determination of rate limiting processes and thus pave the way toward developing the most promising strategies to prevent and control aggregate formation in living systems.

## Author Contributions

GM and DK wrote the paper. GM produced the figures. GM, TK, and DK edited the paper. All authors contributed to the article and approved the submitted version.

## Funding

DK holds a Royal Society Professorship (RSRP\R\210003). His work was supported by the UK Dementia Research Institute which receives its funding from UK DRI Ltd., funded by the UK Medical Research Council, Alzheimer's Society and Alzheimer's Research UK. TK thanks the Frances and Augustus Newman Foundation, the Wellcome Trust, and the European Research Council for financial support.

## Conflict of Interest

GM is a data scientist and TK is a co-founder at Wren Therapeutics. The remaining author declares that the research was conducted in the absence of any commercial or financial relationships that could be construed as a potential conflict of interest.

## Publisher's Note

All claims expressed in this article are solely those of the authors and do not necessarily represent those of their affiliated organizations, or those of the publisher, the editors and the reviewers. Any product that may be evaluated in this article, or claim that may be made by its manufacturer, is not guaranteed or endorsed by the publisher.
